# Childhood Bereavement, Adverse and Positive Childhood Experiences, and Flourishing among Chinese Young Adults

**DOI:** 10.3390/ijerph20054631

**Published:** 2023-03-06

**Authors:** Krista P. Woodward, Zhiyuan Yu, Wenyi Chen, Tingting Chen, Dylan B. Jackson, Terrinieka W. Powell, Lin Wang

**Affiliations:** 1 Department of Population, Family, and Reproductive Health, Johns Hopkins Bloomberg School of Public Health, Baltimore, MD 21205, USA; 2 Johns Hopkins School of Nursing, 525N Wolfe Street, Baltimore, MD 21205, USA; 3 Xin Hua Hospital, School of Medicine, Shanghai Jiao Tong University, Shanghai 200240, China; 4 School of Nursing, Shanghai Jiao Tong University, Shanghai 200240, China

**Keywords:** childhood bereavement, childhood grief, adverse childhood experiences, positive childhood experiences, adult flourishing

## Abstract

Childhood bereavement (CB) resulting from a parent or primary caregiver death is associated with a range of adverse outcomes. Little is known about the association between CB and adult flourishing in the context of adverse childhood experiences (ACEs) and positive childhood experiences (PCEs). In a cross-sectional observational study, we examined how ACEs, PCEs, and adult flourishing differs by self-reported CB history among 9468 Chinese young adults (18–35 years), of which 4.3% experienced CB (*n* = 409). Data collection included convenience sampling among university students in Mainland China. Respondents voluntarily completed an online survey between August and November 2020. Descriptive statistics, chi-square tests, and logistic regressions examined frequencies and differences in ACEs, PCEs, and flourishing by the history of CB controlling for a few demographic covariates. Bereaved individuals reported significantly higher ACEs and lower PCEs. The odds of experiencing emotional, physical, and sexual abuse as well as household substance abuse, parental mental illness, and parental incarceration ranged from 2.0–5.2 times higher for bereaved individuals. Bereaved participants also reported significant negative relationships with Flourishing Index (β = −0.35, t = −4.19, *p* < 0.001) and Secure Flourishing Index (β = −0.40, t = −4.96, *p* < 0.001). Consistent with previous research, our findings demonstrate the lasting effects of CB on well-being. We discuss study implications for ACEs and PCEs screening and surveillance as well as grief counseling to promote flourishing among bereaved youth in China and beyond.

## 1. Introduction

### 1.1. Overview

At least 10,000 children lose a parent every day, leaving a minimum of 140 million children parentally bereaved worldwide [1]. The death of a parent or primary caregiver before the age of 18 (hereafter referred to as childhood bereavement [CB]) is known to be one of the most traumatic and challenging experiences for children and adolescents [2,3,4,5,6,7]. CB can remove important social and economic resources from the bereaved offspring and subsequently affect their development and well-being across the life course [6,7]. CB is now commonly recognized as an adverse childhood experience [8]. However, there is limited research on the effects of CB on health and well-being in the context of other adverse childhood experiences (ACEs) and positive childhood experiences (PCEs), particularly across cultural contexts [9]. Thus, the Traumatic and Adverse Childhood Experiences (TRACES+) conceptual model may be an appropriate lens to study CB in the context of other early life experiences [10]. The TRACES+ conceptual model considers the diverse effects of adverse and positive childhood experiences resulting in heterogeneous neurodevelopmental outcomes across pre-existing contextual factors [10]. This is of critical importance given the downstream effects of early neurodevelopmental trajectories on health [11,12]. Furthermore, most research on CB to date has focused on its association with increasing health risks or developing poor health outcomes, less is known about the impact of CB on long-term well-being, such as adult flourishing [5]. The present study examines the co-occurrence of ACEs and PCEs by a history of CB and the association with adult flourishing in a Chinese young adult sample.

### 1.2. Health Burden of CB across Lifespan

CB is considered a traumatic stressor and generates potentially detrimental consequences across multiple domains of health and well-being [3,4,5,6,7,8]. While most bereaved children can adapt to the aftermath without developing clinically diagnosed mental health conditions, CB may put bereaved individuals at higher risk of experiencing poor health outcomes, such as psychiatric morbidities (e.g., depression, anxiety, and suicidality), substance abuse, heart disease, chronic inflammation, and cognitive decline [3,6,12]. Few studies have systematically examined the underlying mechanisms leading to poor health among those exposed to CB. Hypothesized biological mechanisms suggest that elevated, prolonged stress from CB is tied to systemic and neuroinflammation, leading to poor health outcomes [11,12]. Social and behavioral research has found that depression prior to and immediately following the parent’s death as well as caregiver functioning, mediated functional impairment among bereft youth [6,13].

CB is also associated with negative social and relational outcomes such as diminished self-confidence and dissatisfaction with interpersonal relationships [6,14]. This may further distance bereaved youth from informal social supports found among peers and family [15]. Relatedly, youth with a history of CB are more likely to have poorer educational attainment, leading to less career planning, worse employment opportunities, and economic instability throughout adulthood [6,14]. Such cumulative disadvantages have shown intergenerational effects among families with a history of CB [16,17,18]. Therefore, guided by the integrated TRACES+ conceptual model, we aim to better contextualize CB effects across diverse settings and experiences, as it may help explain the persistent nature of CB [18].

### 1.3. CB in the Context of other Early Life Experiences

Little is known about the effects of CB in the context of or in relation to other early life experiences, such as ACEs and PCEs [19]. Previous research found that prior to parental death, bereaved youth had a higher likelihood of experiencing child maltreatment, parental psychiatric disorder (e.g., depression and substance abuse), and household dysfunction compared to non-bereaved [4,6,19]. These contextual or pre-existing factors may exacerbate or mitigate the effects of CB exposure by augmenting either individuals’ or families’ vulnerabilities or strengths, respectively [10]. Therefore, the effects of CB cannot be studied in isolation but must be considered in the context of other early life experiences to understand the potential compounding effects of adversity accumulation [9].

Adverse childhood experiences (ACEs) are potentially traumatic events that occur before age 18 such as experiencing maltreatment, neglect, or witnessing violence as well as parental death [8,20]. It is well documented that ACEs are strongly associated with a host of negative health and wellness outcomes in adulthood and act in a dose-response manner [8,20,21,22,23,24,25]. Over 50% of children are exposed to at least one ACE, and almost 15% experience four or more ACEs worldwide [8,23]. Since parents are essential for providing a sensitive, nurturing environment along with material support for their children, parental death may increase the likelihood of exposure to additional adverse conditions in the household (e.g., parental mental illness and neglectful parenting) or community (e.g., neighborhood poverty and violence) [25,26].

Although ACEs are common and can have lasting effects, not all individuals exposed to ACEs develop poor health and well-being [10]. In fact, positive childhood experiences (PCEs) often co-occur with ACEs and may buffer the deleterious impact of ACEs [27,28,29]. PCEs may include strong parent-child attachment, peer interconnectedness and belonging, perceived safety, and general support [27]. These protective factors in childhood have been strongly associated with resiliency factors and adaptive skills that lead to stronger social and relational health outcomes in adulthood [27,29]. Thus, parental death may not only increase children’s vulnerabilities to experiencing ACEs, but also deprive a child’s opportunity to benefit from the protective effects of responsive parenting and a safe household environment.

Consistent with the life course perspective, the TRACES+ model acknowledges both risk and protective factors that contribute to equifinality (i.e., multiple different risks may lead to the same negative outcomes) and multifocality (i.e., a particular risk may have heterogenous outcomes) across the lifespan [10]. The TRACES+ model also highlights the interaction between traumatic and adverse childhood experiences (e.g., exposure to CB) and historical, pre-existing, and contextual factors (e.g., exposure to child maltreatment, and parental mental illness prior to the loss of primary caregiver) on neurodevelopment and overall health. Therefore, guided by the TRACES+ model, this study examines the longer-term effects of CB in the context of ACEs and PCEs with a focus on adult flourishing as an important indicator of well-being [10].

### 1.4. CB and Adult Flourishing

Multiple studies have demonstrated the effects of ACEs and PCEs accumulation on well-being measures, such as adult flourishing and resilience, and thus warrants a specific focus on the unique effects of CB [23,24]. Most research to date has focused on the deleterious effects of CB on developing poor health outcomes or risky behaviors [30]. It is important to explore flourishing in addition to mental and physical health outcomes, as it may provide a broader view of health [23,31,32,33]. Human flourishing is a multidimensional concept that extends beyond psychological well-being, and it can be defined as “a state in which all aspects of a person’s life are good” [31]. Previous research has identified six domains of flourishing, including emotional health, physical health, meaning and purpose, character strengths, social connectedness, and financial security [31,32,33]. Empirical evidence suggested that high levels of parent-child connection, family connection, and family resilience have been associated with high levels of adult flourishing, and high exposure to childhood adversities was associated with low levels of adult flourishing [22,27,34]. CB is a traumatic life event, but it is not necessarily indicative of negative outcomes in adulthood; in part, because youth can experience post-traumatic growth after parental loss [30]. Therefore, extending scientific inquiry to adult flourishing may help fill gaps in the field’s understanding of the long-term effects of CB. To the authors’ knowledge, no studies to date have investigated the association between CB and adult flourishing specifically.

### 1.5. CB in Chinese Cultural Context

To date, most research on CB has been conducted in the Western context. However, the experiences of bereavement, grief, and mourning are culturally constructed, so results from existing Western studies cannot be simply imposed across different cultural backgrounds [35,36]. In Chinese culture, it is believed that the relationship between the dead and the living is continuous and that maintaining the continued bond is important [35]. There is also an emphasis on expressing remembrance, grief, and emotions through bereavement rituals rather than verbal means [37]. The conceptualization and thus response to death in Chinese culture are shaped by Confucianism, in which filial piety is the fundamental belief and shapes how families in China experience death. For example, Li and colleagues (2005) noted that during the bereavement period, parents at older ages expect support from their children rather than from their friends [38]. Most Chinese people depend on themselves and their family members for bereavement support and rarely seek professional bereavement support or services, which may exacerbate distress among family members [38]. Further, the terms bereavement and grief do not have a direct English-to-Chinese translation, which may hinder Chinese people’s ability to express and seek support for their emotional needs associated with bereavement, particularly for children.

A few existing China-based studies found that CB was associated with depression, loneliness, anxiety, and impaired cognition (e.g., difficulty with orientation, calculation, recall, and language) in adulthood after controlling for sociodemographic variables and existing mental health conditions [11,21,22,39]. To our knowledge, no study has examined CB in relation to other early life experiences and its association with adult flourishing in China.

### 1.6. Current Study

The purpose of this study was to explore the prevalence of self-reported CB and whether exposure to ACEs and PCEs, and adult flourishing differs by CB history among Chinese young adults. The study team hypothesized that, compared to non-bereaved individuals, bereaved individuals would report (1) higher exposure to ACEs and lower PCEs; (2) lower levels of adult flourishing. The findings of this study will begin to fill the critical knowledge gaps about CB in the context of other early life experiences and its relationship with long-term well-being as indicated by flourishing. Finally, we share study implications on public health-based surveillance as well as clinical practice and programs to better support parentally bereaved individuals in the future.

## 2. Materials and Methods

### 2.1. Study Design

This cross-sectional observational study assessed CB in the context of ACEs and PCEs and their associations with flourishing in a sample of young adults in Mainland China. This study was conducted between August to November 2020. Inclusion criteria included: (1) between 18–35 years old, (2) had enrolled in an undergraduate or graduate program at universities in Mainland China, and (3) agreed to participate in this study. Participants were recruited virtually through convenience and snowball sampling. Potential participants were invited via student cohorts’ online groups on WeChat, the most used communication software in Mainland China. These WeChat groups were formed to help university students connect, communicate, and share information but were not specific to young adults who experienced grief in childhood. The authors did not participate in these WeChat groups. The online survey link that contained the measures described below was distributed to these WeChat groups and was completed independently. Survey data collected were stored on an online survey platform that only authorized access to study team members. The measures included in the survey are detailed below. The Shanghai Jiao Tong University School of Nursing ethics review board approved the current study following national and institutional guidelines. A data-sharing agreement was established across institutions following international and national guidelines.

### 2.2. Measures

#### 2.2.1. Childhood Bereavement (CB)

CB was indicated by a positive response (0 = No; 1 = Yes) to the question “Did a parent or legal guardian die before you turned 18 years old?” in the adapted Chinese version of the World Health Organization (WHO) ACE-International Questionnaire (C-ACE-IQ; 40; detailed description follows).

#### 2.2.2. Adverse Childhood Experiences (ACEs)

The adapted Chinese version of the World Health Organization (WHO) ACE-International Questionnaire (C-ACE-IQ) was used to measure ACEs (40; Chinese version available by request; English version available to download at https://www.who.int/publications/m/item/adverse-childhood-experiences-international-questionnaire-(ace-iq) 24 February 2023). Twelve categories of childhood adversities were assessed in the C-ACE-IQ, including abuse, neglect, household dysfunctions, bullying, and community violence. Responses options include binary answers (i.e., “Yes” or “No”) or frequency answers (e.g., “Many times,” “A few times,” “Once,” Or “Never”). Based on the scoring recommendation of the original WHO ACE-IQ questionnaire (2016), each ACE category was dichotomized into non-exposure (scored 0) and exposure (scored 1). Thus, the cumulative ACEs score ranges from 0–12, with higher scores indicating higher reported exposure to ACEs. Good content validity (scale content validity index [S-CVI] = 0.89) and test-retest reliability (intraclass correlation [ICC] =  0.88) of the C-ACE-IQ has been demonstrated in a sample of 566 Chinese university students [40].

#### 2.2.3. Positive Childhood Experiences (PCEs)

The Chinese version of the Positive Childhood Experiences Scale (C-PCEs) was used to measure PCEs [41]. This 9-item, self-reported scale asked respondents, before the age of 18, how often or how much they: (1) felt able to talk to their family about feelings; (2) felt their family stood by them during difficult times; (3) felt safe and protected by an adult in their home; (4) felt their family relationships are harmonious; (5) felt treated fairly at school; (6) felt a sense of belonging in school; (7) felt supported by friends; (8) had at least two non-parent adults who took a genuine interest in them; and (9) received affirmation, encouragement, or support. Response options for these nine items of PCEs were “Never,” “Rarely,” “Sometimes,” “Often,” and “Very often.” Responses to each PCEs item were dichotomized into 0 (“Never or rarely”) or 1 (“Very often, often, or sometimes”). The total score of the scale ranges from 0–9, with higher scores indicating higher exposure to PCEs. The C-PCEs Scale demonstrated good content validity (Cronbach’s α = 0.72) and test-rest reliability (intraclass correlation [ICC] = 0.75) in this study sample. Factor analysis confirmed the C-PCEs comprised two subdimensions, household (items 1–4) and community (items 5–9) PCEs [41]. Two Eigenvalues greater than 1.0 (Factor 1: 3.005 explains 33.38% of the variance and Factor 2: 1.329 explains 14.76 % variance).

#### 2.2.4. Adult Flourishing

The Chinese version of the Flourishing Measure was used to assess flourishing which has been used in large-scale cross-cultural studies [23,31,42,43]. This translated measure assesses six domains of flourishing: (1) happiness and life satisfaction, (2) mental and physical health, (3) meaning and purpose, (4) character and virtue, (5) close social relationships, and (6) financial and material stability. Each domain comprises two Likert scale questions, with each question’s response ranging from 0–10 (e.g., 0 = Extremely disagree and 10 = Extremely agree). In this study two summary indices, the “Flourish Index” (FI) and the “Secure Flourish Index” (SFI), were generated to reflect participants’ level of flourishing as a continuous variable. The “Flourish Index (FI)”, the average score of ten questions in the first five domains, reflects “core constituents of flourishing” [31]. The “Secure Flourish Index (SFI)”, the average score of twelve questions from all six domains, reflects flourishing over an extended period since the financial and material stability domain is seen as “a means of sustaining these five core constituents over time” [31]. Both indices’ average score ranges from 0–10, with higher scores indicating higher levels of perceived flourishing. In a previous study with Chinese clothing supply chain workers, the Chinese version of FI and SFI had shown good internal consistency (Cronbach’s α = 0.88 and 0.81, respectively) [42]. The internal consistency of FI and SFI in this study sample was 0.91 and 0.89, respectively.

### 2.3. Covariates

Demographic characteristics including self-reported gender (female vs. male), age (18–35 years), year in university (freshman, sophomore, junior, senior, and graduate school), and marital status (single, married or cohabitating, divorced, separated, widowed, and other) were also included in analyses. These covariates were selected based on previous empirical studies on flourishing [44,45].

### 2.4. Data Analysis

Statistical analyses were performed using SPSS 27.0. Descriptive statistics were used to describe study variables. Expectation Maximization (EM) procedure was used to assess missing data patterns, and results confirmed that data were missing at random. SPSS performs listwise deletion of missing data, meaning, the entire case will be excluded if there are any missing values for any variables included in the analysis. There were 139 participants who had missing data on CB history, which accounted for 1.5% of the total sample size (*n* = 9468), so we did not impute any missing values. The two sample *t*-tests and Fisher’s exact test were used to examine whether there were any differences in demographic characteristics (i.e., age, gender, year in university, and marital status) between the CB missing group and the CB non-missing group.

Chi-square analyses were conducted to examine unadjusted relationships between young adults’ self-reported CB history and their exposures to ACEs and PCEs and levels of flourishing. Univariate and multivariate logistic regression models were used to investigate the unadjusted and adjusted relationships between young adults’ self-reported CB history and their exposures to each ACEs and PCEs type. In multivariate logistic models that assess the relationship between CB history and each ACE type, gender, and PCEs sum score were controlled. In multivariate logistic models that assess the relationship between CB history and each PCE type, gender was controlled. Simple and multiple linear regression analyses were used to investigate the unadjusted and adjusted relationships between CB history and level of flourishing. In the multiple linear regression model, gender, age, year in university, marital status, and PCEs sum score were controlled. The level of statistical significance was set at alpha = 0.05.

## 3. Results

### 3.1. Participant Characteristics and Childhood Bereavement

Table 1 lists the detailed demographic characteristics of participants. The analytic sample included 9468 young adults with a mean age of 20.1 years (SD = 1.7). Most participants were undergraduate students (96.4%) and single (79.8%) and three-quarters of the participants were female (75.3%). Among this sample, 409 (4.3%) self-reported exposure to the death of a mother, father, or guardian before 18 years.

The two sample *t*-tests and Fisher’s exact test showed that only marital status was significantly different between the CB-missing and CB non-missing groups (*p* = 0.033). Compared to the CB missing group, the CB non-missing group had a higher proportion of people who reported “other” marital status (13.5% vs. 9%). Compared to the CB missing group, the CB non-missing group had a lower proportion of people who “refused to answer” their marital status (4.5% vs. 11.5%). There are no other significant differences in other categories of marital status (i.e., married, co-habitat, divorced or separated, single, widowed).

### 3.2. ACEs, PCEs, and Adult Flourishing by Bereavement History

Table 2 compares the types of exposure to ACEs by CB history. The mean cumulative ACEs scores among the bereaved sample were significantly higher than those among the non-bereaved sample (means = 2.56 [SD = 2.00, range 1–12] vs. 1.03 [SD = 1.36, range 0–10]; Chi-square = 559.84, *p* < 0.001). More than 20% reported exposure to four or more ACEs among the bereaved sample, compared to about 6% among the non-bereaved sample. Univariate and multivariate logistic regression models both showed that the bereaved sample also had significantly higher odds of exposure to each ACE type.

Table 3 compares types of exposure to PCEs by CB history. The mean cumulative PCEs scores among the bereaved sample were significantly lower than those among the non-bereaved sample (means = 7.97 [SD = 1.77, range 0–9] vs. 8.40 [SD = 1.20, range 0–9]; Chi-square = 52.64, *p* < 0.001). About 85% reported exposure to 7–9 PCEs among the bereaved samples compared to about 93% among the non-bereaved sample. Both univariate and multivariate logistic regression models showed that the bereaved sample had significantly lower odds of exposure to each PCE type except for being treated fairly at school (PCE item 5) and affirmation (PCE item 9).

Table 4 compares six domains and indices of flourishing by CB history. All six domains and indices of flourishing among the bereaved sample were significantly lower than those among the non-bereaved sample. Results from simple linear regression analysis showed that CB history had significantly negative relationships with Flouring Index (FI—mean of the first five domains; β = −0.35, t = −4.19, *p* < 0.001) and Secure Flourishing Index (SFI—mean of all domains; β = −0.40, t = −4.96, *p* < 0.001). Results from multiple linear regression analysis showed that CB history no longer had significant negative relationships with FI (β = −0.08, t = −1.06, *p* = 0.290) and SFI (β = −0.13, t = −1.75, *p* = 0.08) after controlling for demographic variables (i.e., gender, age, year in university, marital status) and PCE sum score. However, in the multiple linear regression model, age (FI: β = 0.04, t = 2.93, *p* = 0.003; SFI: β = 0.03, t = 2.39, *p* = 0.017) and PCE sum score (FI: β = 0.50, t = 38.19, *p* < 0.001; SFI: β = 0.49, t = 38.42, *p* < 0.001) still had significant positive relationships with FI and SFI.

## 4. Discussion

This study explored the prevalence of CB in the context of other ACEs and PCEs and its association with adult flourishing in a sample of Chinese young adults. We found that approximately 4.3% of participants in our sample self-reported CB exposure. Among those with CB exposure, participants also reported higher exposure to ACEs, lower exposure to PCEs, and a lower level of adult flourishing compared to their non-bereaved counterparts. This study is innovative as it explored the effects of CB beyond poor health outcomes or risky behaviors by focusing on adult flourishing, which provides a more comprehensive understanding of wellbeing [24,27]. Existing literature on CB has explored the sociodemographic risks among bereaved children, such as poverty, but few have explored CB in the context of ACEs and PCEs which may either exacerbate or mitigate the negative relationship of CB on bereaved children [6,9]. Furthermore, this study focuses on CB in a non-Western context (i.e., China) thereby broadening our cross-cultural knowledge of CB.

Nearly all bereaved participants (98.5%) reported at least one ACE and 21.3% reported 4 or more ACEs. Notably, the odds of experiencing emotional, physical, and sexual abuse as well as household substance abuse, parental mental illness, and parental incarceration ranged from 2–5.2 times higher for bereaved compared to non-bereaved individuals. Similar to previous research findings, the current study also captured how early parental death is associated with parental poor mental and physical health, substance abuse, and more [4,6]. Higher odds of exposure to ACEs in bereaved participants might be explained by the disruption in family living arrangements following parental death. CB may lead to harmful caretaking and cohabitation circumstances, such as living non-biological adults which is a known risk factor for child maltreatment and abuse [46].

Alternatively, the higher odds of ACEs exposure may also be explained by the sudden lacking material and financial support resulting from the parental death, particularly if the deceased parent was the primary source of household income. Such resources or financial deprivation may put undue stress on surviving parents and/or caretakers and thrust families into poverty who were already living on the “edge”. Research has shown that resource deprivation (e.g., food or housing insecurity) is associated with increased physical violence upon young children [25,26,47]. Such stress may also lead the surviving parent to maladaptive coping strategies for grief and distress, such as parental incarceration and substance abuse, which may in turn expose their children to additional childhood adversities.

The observed high co-occurrence of adversities among bereaved children underscores the need to conduct regular ACE screenings in healthcare settings. These targeted prevention screenings may help mitigate the effects of bereavement and prevent exposure to other adversities [48,49]. Universal screening for ACEs in clinical settings among bereaved individuals may mitigate additional harm by allowing providers to connect patients with supportive services outside of their medical homes. Several efficient, effective, and feasible screening tools exist to help providers support bereaved youth in this capacity [49].

In addition, population-based surveillance systems that collect data on the duration, frequency, and severity of ACEs are critical for devising effective data-driven prevention and intervention efforts as well as allocating resources to populations in greatest need, such as bereaved children and families [50]. Examples of population-based surveillance include the Behavioral Risk Factor Surveillance System (BFRSS) which has recently included bereavement items along with other ACEs in the US. Similarly, the China Health and Retirement Longitudinal Survey (CHARLS) has provided data on ACEs among individuals aged 45 or above. Studies using the CHARLS dataset have found the intergenerational relationship between parents’ ACEs on their children’s behavioral problems and poor physical and mental health [51]. However, China currently lacks infrastructure that can systematically collect prospective or proximal data for current prevention efforts that would reduce recall bias associated with retrospective studies with adults [51]. As recommended by Anderson and colleagues, one potential surveillance mechanism may be to leverage administrative data, such as a national census [50].

We found that bereaved young adults were not only more likely to be exposed to ACEs, but also less likely to have PCEs compared to non-bereaved. Specifically, they were less likely to feel that their family stood by them during a difficult time or feel protected by an adult in their home. Reduction in surviving caregivers’ parenting capacity can cause bereaved children to experience “double jeopardy” whereby bereaved youth also suffer the symbolic or temporary loss of the surviving parent [52]. In a recent conceptual model of ACEs, Sheridan and McLaughlin (2014) proposed that there were two primary dimensions of early life experiences that generate differential impact on developmental outcomes including threat (presence of atypical experiences that threaten one’s physical integrity, e.g., abuse) and deprivation (absence of expected cognitive and social input, e.g., neglect) [53]. Results from our study suggest that CB may fall in the domain of complex exposure which refers to experiences that involve aspects of both threat and deprivation [53]. However, our study did not measure the nuances of CB, for example, the nature and timing of the death, the nature of the relationship to the deceased caregiver, and the temporal sequence of the parental death in relation to other childhood experiences measured. These are important aspects that should be integrated into future studies of CB, especially if the deceased caregiver was the main source of PCEs, since their absence may expose children to more threat dimensions of ACEs (e.g., violence and abuse). On the contrary, if the deceased caregiver is the perpetrator of violence or abuse, the removal of such figures from the child may reduce their exposure to environmental threats.

As we hypothesized, the mean flourishing scores (i.e., six domains and two indices) among the bereaved sample were significantly lower than their non-bereaved counterpart. Results from multiple linear regression analysis showed that CB history did not have significant relationships with FI and SFI mean scores after controlling for PCE score and demographic variables (i.e., gender, age, year in university, marital status). These findings suggested that despite the association of CB with lower adult flourishing, PCEs can set a positive trajectory for adult flourishing among bereaved youth. Our study findings are consistent with empirical evidence that PCEs had positive correlations with all flourishing indices and domains amidst other childhood adversities [24]. Interventions promoting PCEs, in addition to mitigating ACEs, have been associated with healthier development and higher levels of adult flourishing [27,28]. Healthcare and education professionals (e.g., teachers, school counselors) who work closely with bereaved children and families can promote PCEs by establishing positive, supportive relationships with children and the surviving caregiver, educating families regarding the importance of positive parenting, and creating a safe environment for children and the surviving caregiver to grief and build adaptive coping skill [6,29]. Therefore, grief programs may be more effective if they promote healthy relationships between the child and surviving parent/caregiver and other family members [6,54,55,56].

The prevalence of CB in our sample is comparable to the U.S. (2.3% to 11% depending on the sample and measurement used) and other Western countries such as Sweden and the United Kingdom (4–5%) [57]. Despite the similar prevalence, professional grief and public sources of bereavement support are scarce in China [58]. Thus, bereaved children and families in China are less supported to navigate the process of bereavement and grief compared to those in countries with such existing infrastructure and resources. Formalized bereavement programs—those led by certified healthcare professionals—have been shown to be effective by promoting adaptive coping skills and healthy behaviors, strengthening parent-child bonds, and enhancing emotional regulation [6,54,55,56,59]. Children who participate in formalized grief programming have fewer depressive episodes and better mental health overall [59]. However, lacking mental health infrastructure for bereaved youth in schools, religious institutions, and community service organizations, has led to social exclusion, bullying, and difficult friendships [5]. Educational settings such as schools where children spend the majority of their time and developing important social relationships could be valuable places for CB intervention [5]. Given the compulsory nine-year education requirement in China, school systems could serve as the “first responders” to recognize distress in bereaved children by assessing other adversities, needs, and strengths among children exposed to CB and connecting them to other services [54,59,60]. School systems can consider strategies such as providing teachers, school nurses, and administrators with bereavement and ACEs training, implementing death education in the school curriculum, and promoting a culture that allows students to talk about death and grief [60,61,62]. In future studies, researchers could develop or culturally adapt existing school-based bereavement interventions to meet the needs of children in China in collaboration with parents and teachers [60,61,62,63].

### Limitations

The current study is not without limitations. First, we used a convenience sample of undergraduate and graduate students in this study which represent a relatively privileged group in Mainland China. Even though our results may have limited generalizability to the broader Chinese population, it is important to note that the majority of Chinese young adults attend higher education beyond high school as of 2022 [64]. However, the fact that the bereaved sample in our study reported significantly higher odds of exposure to childhood maltreatment and household dysfunction highlights the need to explore CB in the context of other ACEs in populations with diverse socioeconomic backgrounds, particularly those living in low-resourced settings in future studies. Second, this is a cross-sectional study that relied on retrospective self-report measures. Therefore, we are not able to infer casual relationships and establish the temporality of the event sequence, that is, whether the loss of the primary caregiver/guardian occurred before or after the reported ACEs and PCEs. The self-report nature of data collection is also susceptible to social desirability, shared method bias and recall bias [65]. Third, it is important to note that the current study did not include potential sociodemographic confounders such as parent death contextual details, ethnicity, family income, and parental education, which are closely related to CB and adult flourishing [7]. Given the risk of omitted variable bias that this poses, future research should seek to replicate these findings with alternative data sources that can account for these factors. Lastly, the data collection phase of the study occurred during the COVID-19 pandemic, which may have magnified grief recall and lowered adult flourishing scores. A few preliminary studies have found that flourishing and well-being did significantly decrease during COVID-19 among university students [66]. Thus, future studies may replicate the investigation post-COVID period. Despite these limitations, the current study has provided important descriptive evidence for future research to explore moderators and underlying mechanisms of CB on adult flourishing.

Future studies should consider using a prospective, longitudinal design that uses multi-reporter measures on CB, ACEs, and PCEs to gain a more nuanced and in-depth understanding of how timing and characteristics of CB interact with types and severity of ACEs and PCEs to shape health and other outcomes. Additional research is needed to understand mechanisms that may be driving the relationship between CB and poor short- and long-term health outcomes, especially in the context of other concomitant adversities experienced within the family and broader social structures [12,17]. In addition, researchers may consider conducting both quantitative and qualitative studies to gain a better understanding of the characteristics and impacts of CB in the general Chinese population [67]. As such, there is a great need to develop culturally sensitive, trauma-informed bereavement interventions and evaluate the efficacy of these programs in China and globally. This research would enhance understanding of barriers to accessing formalized bereavement services, such as stigma, self-reliance, limited availability, and how informal social supports through social networks supplement care for CB throughout development [67]. Given the dearth of CB research in China, it would be beneficial to study its effects on population health and well-being to justify increased funding and attention to surveillance systems and trauma-informed grief support services.

## 5. Conclusions

The loss of a parent or primary caregiver is a significant childhood adversity with implications for adult health and well-being. The current study extends previous research by exploring how co-occurring ACEs and PCEs among bereaved children influence overall well-being as indicated by flourishing. The need for research on CB risk factors and health impacts is even more pressing after the COVID-19 pandemic, especially across cultural contexts [68]. The prevalence of CB has exponentially grown during the COVID-19 pandemic, leaving as many as 10.5 million children orphaned from COVID-19-related complications [68]. Therefore, the public health field may observe a sizable increase in overall adversity among families in the coming years above and beyond their grief. By studying the cooccurrence of ACEs among bereaved youth, we may be one step closer to addressing the multifactorial nature of CB experienced during the COVID-19 pandemic and beyond.

## Figures and Tables

**Table 1:** Demographic characteristics of the sample.

	Full Sample * (N = 9468)	Bereaved Participants (*n* = 409)	Non-Bereaved Participants (*n* = 8920)
Age (in years)			
Range	18–35	18–30	18–35
Mean (SD)	20.1 (1.7)	20.2 (1.6)	20.0 (1.6)
Gender, *n* (%)			
Female	7129 (75.3)	309 (75.6)	6724 (75.4)
Male	2244 (23.7)	98 (24.0)	2106 (23.6)
Missing	95 (1.0)	2 (0.5)	90 (1.0)
Year in university, *n* (%)			
Freshman	2146 (22.7)	93 (22.7)	2028 (22.7)
Sophomore	2652 (28.0)	106 (25.9)	2505 (28.1)
Junior	2986 (31.5)	122 (29.8)	2819 (31.6)
Senior	1342 (14.2)	76 (18.6)	1240 (13.9)
Graduate	259 (2.7)	8 (2.0)	249 (2.8)
Missing	83 (0.9)	4 (1.0)	79 (0.9)
Marital status, *n* (%)			
Single	7554 (79.8)	321 (78.5)	7125 (79.9)
Married or cohabitate	107 (1.1)	6 (1.5)	100 (1.1)
Other **	1807 (13.6)	82 (20.1)	1695 (19)
ACEs by levels of exposure, *n* (%)			
0 ACE	4151 (43.8)	6 (1.5)	4092 (45.9)
1–3 ACEs	4662 (49.2)	316 (77.3)	4265 (47.8)
4–12 ACEs	655 (7.0)	87 (21.3)	563 (6.3)
PCEs by levels of exposure, *n* (%)			
0–3 PCEs	110 (1.2)	15 (3.7)	92 (1.0)
4–6 PCEs	650 (6.9)	48 (11.7)	583 (6.5)
7–9 PCEs	8708 (92.0)	346 (84.6)	8245 (92.5)

Note. * The full sample included participants who did not report on CB status. ** Other includes missing, divorced, separated, widowed, or another marital status.

**Table 2:** ACEs types of exposure by adult self-reported childhood bereavement history.

ACEs by Types, *n* (%)	Bereaved Participants (*n* = 409)	Non-Bereaved Participants (*n* = 8920)	Chi-Square (*p*-Value)	Odds Ratio (*p*-Value)	Adjusted Odds Ratio ^a^ (*p*-Value)
Emotional Neglect	178 (43.5)	2905 (32.6)	23.21 (<0.001)	1.65 (<0.001)	1.39 (0.005)
Household violence	118 (28.9)	1810 (20.3)	18.23 (<0.001)	1.62 (<0.001)	1.33 (0.023)
Sexual Abuse	74 (18.1)	872 (9.8)	30.05 (<0.001)	2.06 (<0.001)	1.76 (<0.001)
Community violence	57 (13.9)	707 (7.9)	18.34 (<0.001)	1.87 (<0.001)	1.56 (0.005)
Emotional Abuse	53 (13.0)	608 (6.8)	22.72 (<0.001)	2.05 (<0.001)	1.48 (0.025)
Family mental illness	39 (9.5)	197 (2.2)	88.88 (<0.001)	4.82 (<0.001)	3.98 (<0.001)
Incarcerated household member	38 (9.3)	174 (2.0)	97.17 (<0.001)	5.24 (<0.001)	4.45 (<0.001)
Physical Abuse	34 (8.3)	370 (4.1)	16.60 (<0.001)	2.11 (<.0001)	1.50 (0.048)
Family substance abuse	30 (7.3)	189 (2.1)	47.67 (<0.001)	3.72 (<0.001)	3.00 (<0.001)
Physical Neglect	22 (5.4)	287 (3.2)	6.05 (0.014)	1.74 (<0.001)	1.38 (0.179)
Parental separation/divorce	95 (9.3)	294 (3.6)	70.31 (<0.001)	2.70 (<0.001)	2.39 (<0.001)
Bullying	15 (3.7)	112 (1.3)	17.54 (<0.001)	3.05 (<0.001)	1.87 (0.044)

Note. ACEs = Adverse childhood experiences. ^a^ controlled for covariates gender and PCEs total score.

**Table 3:** PCEs types of exposure by adult self-reported childhood bereavement history.

PCEs by Types, *n* (%)	Bereaved Participants (*n* = 409)	Non-Bereaved Participants (*n* = 8920)	Chi-Square (*p*-Value)	Odds Ratio (*p*-Value)	Adjusted Odds Ratio ^a^ (*p*-Value)
1. Able to talk to your family about feelings	313 (76.53%)	7751 (87%)	36.83 (<0.001)	0.49 (<0.001)	0.49 (<0.001)
2. Family stood by you during difficult times	357 (87.29%)	8420 (94.6%)	38.72 (<0.001)	0.39 (<0.001)	0.39 (<0.001)
3. Feel safe and protected by an adult in your home	363 (89.41%)	8557 (96.09%)	43.11 (<0.001)	0.34 (<0.001)	0.34 (<0.001)
4. Family relationships are harmonious	378 (93.56%)	8663 (97.4%)	20.79 (<0.001)	0.39 (<0.001)	0.39 (<0.001)
5. Treated fairly at school	389 (95.34%)	8603 (97.02%)	3.72 (0.05)	0.63 (0.056)	0.63 (0.058)
6. Feel a sense of belonging in school	337 (82.6%)	7799 (87.8%)	9.69 (0.002)	0.66 (0.002)	0.66 (0.002)
7. Feel supported by friends	386 (94.38%)	8648 (97.07%)	9.60 (0.002)	0.51 (0.002)	0.51 (0.002)
8. Have at least 2 nonparent adults who took a genuine interest in you	355 (87.22)	8049 (90.73%)	5.62 (0.02)	0.70 (0.018)	0.70 (0.017)
9. Receive affirmation, encouragement, or support	380 (93.37%)	8462 (95.07%)	2.37 (0.12)	0.73 (0.125)	0.73 (0.119)

Note. PCEs = Positive childhood experiences. ^a^ controlled for covariates gender.

**Table 4:** Adulthood flourishing by self-reported childhood bereavement history.

	Full Sample (N = 9468)	Bereaved Participants (*n* = 409)	Non-Bereaved Participants (*n* = 8920)	
	Mean (SD)	Mean (SD)	Mean (SD)	Chi-Square (*p*-Value)
Domain 1: Happiness and life satisfaction	6.93 (1.65)	6.47 (2.16)	6.94 (1.95)	56.38 (<0.001)
Domain 2: Mental and Physical Health.	6.87 (1.61)	7.05 (1.97)	7.53 (1.78)	66.31 (<0.001)
Domain 3: Meaning and Purpose.	6.91 (1.96)	6.67 (2.05)	6.92 (1.91)	44.04 (<0.001)
Domain 4: Character and Virtue	7.50 (1.80)	6.44 (2.11)	6.69 (1.87)	43.70 (0.002)
Domain 5: Close Social Relationships	6.90 (1.92)	6.43 (2.10)	6.72 (1.98)	30.92 (0.056)
Domain 6: Financial and Material Stability	6.67 (1.89)	5.91 (2.55)	6.59 (2.41)	49.37 (<0.001)
Flourishing Index (FI)	6.70 (1.99)	6.61 (1.78)	6.96 (1.63)	165.83 (<0.001)
Secure Flourishing Index (FSI)	6.55 (2.42)	6.49 (1.72)	6.90 (1.59)	193.92 (<0.001)

## Data Availability

Due to the sensitive nature of the questions asked in this study, survey respondents were assured raw data would remain confidential and would not be shared.
